# Clinical outcome and prognostic factors associated with invasive pulmonary aspergillosis: An 11-year follow-up report from Taiwan

**DOI:** 10.1371/journal.pone.0186422

**Published:** 2017-10-19

**Authors:** Kuo-Shao Sun, Ching-Fang Tsai, Solomon Chih-Cheng Chen, Wan-Chun Huang

**Affiliations:** 1 Division of Pulmonary and Critical Care Medicine, Ditmanson Medical Foundation Chia-Yi Christian Hospital, Chiayi City, Taiwan; 2 Chung-Jen Junior College of Nursing, Health Sciences and Management, Chiayi City, Taiwan; 3 Department of Medical Research, Ditmanson Medical Foundation Chia-Yi Christian Hospital, Chiayi City, Taiwan; 4 Taipei Medical University, Taipei City, Taiwan; 5 Department of Pediatrics, Ditmanson Medical Foundation Chia-Yi Christian Hospital, Chiayi City, Taiwan; J Craig Venter Institute, UNITED STATES

## Abstract

**Background:**

Invasive pulmonary aspergillosis (IPA) has high mortality rate but prognostic factors are not well established. The aim of our study was to evaluate the trend in in-hospital mortality over a period of 11 years and identify factors affecting the clinical outcomes of patients with IPA.

**Method:**

We conducted a nationwide inpatient population study using data from the Taiwan National Health Insurance Research Database. A total of 407 IPA patients from 2002 to 2012 were included in the study. Differences in demographics, comorbidities, and treatment were evaluated between in-hospital death group and survival group. Multivariate analysis was also performed to identify risk factors for mortality.

**Result:**

Male patients represented 63.14% of the patients (n = 257) and the mean age was 53.15 ± 20.93 years. Hematological cancer (n = 216, 53.07%) and diabetes mellitus (n = 75, 18.43%) were the most common underlying conditions. The overall case fatality rate was 30.22% with female slightly higher then male (32.67% versus 28.79%). The in-hospital case fatality rate increased since 2002 and peaked in 2006. It then declined over time with an in-hospital mortality of 25% in 2012. The in-hospital death group had a higher intubation rate (p<0.0001), a longer ICU stay (p = 0.0062), higher percentages of DM (p = 0.0412) and COPD (p = 0.0178), and a lower percentage of hematological cancer (p = 0.0079) as compared to survivor. The in-hospital death group was more likely to have steroid treatment (p<0.0001), develop acute renal failure (p<0.0001) and other infectious diseases (p = 0.0008) during hospitalization. Multivariate analysis identified female gender, older age (≥ 65 years old), intubation, bone marrow transplantation, acute renal failure, other infectious diseases and steroid use as predictive factors for mortality.

**Conclusion:**

The present study shows the trend in mortality among patients with IPA over an 11-year period. Female gender, older age, intubation, bone marrow transplantation, acute renal failure, other infectious diseases and steroid use were identified as risk factors for mortality.

## Introduction

Invasive aspergillosis (IA) is a common invasive mold infection that mostly affects immunocompromised patients. Most cases of IA have pulmonary involvement and often lead to severe pneumonia with respiratory failure requiring intubation and mechanical ventilation [1, 2]. The incidence of invasive pulmonary aspergillosis (IPA) has been increasing over the years [3], and patients with pre-existing conditions such as end-stage renal disease (ESRD), diabetes mellitus (DM), chronic obstructive pulmonary disease (COPD), and long-term steroid use are more susceptible than previously suspected [4].

The high mortality rate associated with IA remains an important issue, with studies reporting a crude mortality ranging from 35.6% to as high as 70% in critically ill patients [1, 2, 5–10]. Though new diagnostic tools such as the galactomannan (GM) test, newer anti-mold medicines and increased disease awareness in non-hematological patients have been shown in studies to be effective in improving early diagnosis and clinical outcomes [11–13], it is unclear whether mortality has been consequently reduced over time. In the study done by Kim et al., crude mortality rates were significantly lower in 2005–2006 compared with 2000–2004 (31.2% versus 39.0%) [6]. Nevertheless, another population-based analysis illustrated no obvious change in mortality rates from 2001 to 2010 [14].

Identification of predictors for mortality, in addition to newer diagnostic methods and anti-mold treatment, may help recognize patients with higher mortality rate who could benefit from more aggressive therapies and lead to better clinical outcomes [5]. However, previous studies have been inconsistent in identifying prognostic factors. A single center study of IA by Hsiue et al. reported that steroid use correlated with poorer survival [15], while Iqbal et al. found no significant association between steroid exposure and morality in Pakistani patients with IPA [16]. The latter authors identified respiratory failure, DM, and longer length of hospital stay to be independent factors that predicted poor outcome. A large-scale international multicenter study conducted by Tacoone et al. investigated ICU patient with IA and found that older age, bone marrow transplant, higher sequential organ failure assessment (SOFA) score, mechanical ventilation use and requirement for renal replacement therapy were predictors of a poor outcome [1]. Another multicenter study by Porpon et al. evaluated the epidemiology of invasive mold infection in five Asian countries and concluded that disseminated diseases, rheumatologic condition and higher GM levels were predictors of mortality [8]. Since there is no overall consensus, it is necessary to have more evidence regarding prognostic factors to identify patients with IPA who are at a higher risk of mortality.

Therefore, the aim of this study is to describe the trend in mortality among Taiwanese patients with IPA over an 11-year period and to identify the associated prognostic factors based on data from the national health insurance database.

## Materials and methods

### Data sources

The data for this population-based case-control study were retrieved from the Taiwan National Health Insurance Research Database (NHIRD). The NHIRD is a comprehensive medical claims database that contains demographic information such as sex and date of birth as well as health care information such as the dates of visits and admissions, records of prescriptions, and up to five discharge diagnoses. The International Classification of Diseases, Ninth Revision, Clinical Modification (ICD-9-CM) codes were used for disease diagnosis. The Ethics Review Board of the Ditmanson Medical Foundation Chia-Yi Christian Hospital in Taiwan approved this study. Because all patient identification numbers in NHIRD were scrambled before data release to protect confidentiality, no informed consent was necessary.

### Study subjects

We identified all first-admitted patients with the diagnosis of IPA (ICD-9-CM codes 484.6) between 2002 and 2012 who were treated with either oral and/or intravenous antifungal therapy, which included voriconazole, posaconazole, micafungin, itraconazole, caspofungin, anidulafungin and amphotericin B during their hospitalization. Only patients who received at least 3 days of antifungal therapy were selected to ensure the accuracy of the IPA diagnosis. This definition of IPA was applied in our previous study [3]. We defined patients who died during the hospitalization (in-hospital death) as the case group, and patients who were discharged from the hospital (survival) as the control group.

### Definition of characteristics, comorbidity, and drug use

The demographics, comorbidity, and drug use among patients were assessed in this study. Age was categorized into four groups: 0 to 18, 19 to 49, 50 to 64, and over 65 years old. The comorbidities included DM (ICD-9-CM code 250), ESRD (ICD-9-CM codes 585.5, 585.6 combined with ICD-9-CM procedure codes 39.95, 38.95), liver cirrhosis (ICD-9-CM code 571), COPD (ICD-9-CM codes 491, 492, 496 and age over 40 years), asthma (ICD-9-CM code 493), bronchiectasis (ICD-9-CM code 494), tuberculosis (ICD-9-CM codes 011, 012, 018), acquired immune deficiency syndrome (AIDS, ICD-9-CM code 042), autoimmune disease (ICD-9-CM codes 710.0–710.4, 714), solid organ cancer (ICD-9-CM codes 140–195), hematological cancer (ICD-9-CM codes 204–208), and transplantation classified into bone marrow (ICD-9-CM codes V428.1-V428.2) and organ transplantation (ICD-9-CM codes V420, V421, V426, V427, V428.3-V428.4). We also investigated the impact of acute renal failure (ICD-9-CM code 584.5–584.9), other infectious diseases excluding tuberculosis, AIDS, aspergillosis (ICD-9-CM codes 001–010, 013–016, 019.0–117.2, 117.4–139.8, 320–326, 480.0–484.5, 484.7–487.8), and medications such as voriconazole and steroids during the hospitalization.

### Statistical analysis

A t-test and chi-square test were used to evaluate the differences in patient characteristics, comorbidity, and drug used between the in-hospital death group and the survival group. The in-hospital mortality rate was calculated and plotted by year and age group. To investigate the risk of in-hospital mortality in patients with IPA, we used a logistic regression model to calculate the odds ratios (ORs) and 95% confidence intervals (CIs). All statistical analyses were performed using SPSS software, Version 21 of the SPSS System for Windows (version 21.0; IBM Corporation, Somers, NY, USA). A two-tailed P-value < 0.05 was considered statistically significant.

## Results

A total of 407 cases were identified from our database. Patient characteristics are shown in Table 1. The mean age was 53.15 ± 20.93 years, and male patients represented 63.14% of the patients (n = 257). A total of 147 (36.12%) patients were admitted to the intensive care unit (ICU), and 136 (33.42%) patients were intubated. The median overall duration of hospital stays was 35 days and was 11 days for ICU stays. Chest computed tomography was performed in the majority of the patients (n = 300, 73.71%). Bronchoscopy and the GM test were performed in 31.45% and 30.71% of the patients, respectively. The most common comorbid conditions are hematological cancer (n = 216, 53.07%) and diabetes mellitus (n = 75, 18.43%). COPD (n = 41, 10.07%) and asthma (n = 25, 6.14%) were the most common underlying lung diseases. In terms of medications, steroids were prescribed in 331(81.33%) patients and voriconazole was used in 282 (69.29%) patients as either mono-therapy or part of a combination therapy.

**Table 1:** Demographic characteristics, clinical features, and resource utilizations of patients with invasive aspergillosis pneumonia (N = 407).

Variables -Number (%)	Total (N = 407)	Survival (N = 284)	In-hospital death (N = 123)	P value
Gender				
Female	150 (36.86)	101 (35.56)	49 (39.84)	0.4118
Male	257 (63.14)	183 (64.44)	74 (60.16)	
Age, years				
0–18	34 (8.35)	31 (10.92)	3 (2.44)	<0.0001
19–49	121 (29.73)	90 (31.69)	31 (25.20)	
50–64	117 (28.75)	87 (30.63)	30 (24.39)	
65+	135 (33.17)	76 (26.76)	59 (47.97)	
mean±SD	53.15±20.93	50.46±21.01	59.36±19.46	<0.0001
GM test	125 (30.71)	84 (29.58)	41 (33.33)	0.4507
Bronchoscopy	128 (31.45)	75 (26.41)	53 (43.09)	0.0009
Computered tomography	300 (73.71)	207 (72.89)	93 (75.61)	0.5667
Use of intubation and MV	136 (33.42)	45 (15.85)	91 (73.98)	<0.0001
ICU admission	147 (36.12)	59 (20.77)	88 (71.54)	<0.0001
Length of stay, days- Median (IQR)				
Overall	35 (21–54)	35.0 (20.5–52.5)	33.0 (21.0–56.0)	0.4297
In ICU admission	11 (5–22)	8.0 (2.0–18.0)	14 (6.0–24.5)	0.0062
Medical cost, US dollars- Median (IQR)	15045 (7127–27743)	13120 (5983–25836)	19729 (10919–34599)	<0.0001
Comorbidity				
DM	75 (18.43)	45 (15.85)	30 (24.39)	0.0412
ESRD	14 (3.44)	9 (3.17)	5 (4.07)	0.6488
Liver cirrhosis	25 (6.14)	20 (7.04)	5 (4.07)	0.2507
COPD	41 (10.07)	22 (7.75)	19 (15.45)	0.0178
Asthma	25 (6.14)	18 (6.34)	7 (5.69)	0.8029
Bronchiectasis	19 (4.67)	14 (4.93)	5 (4.07)	0.7042
Tuberculosis	40 (9.83)	29 (10.21)	11 (8.94)	0.6931
AIDS	4 (0.98)	2 (0.70)	2 (1.63)	0.5876
Autoimmune disease	27 (6.63)	17 (5.99)	10 (8.13)	0.4248
Cancer				
Solid organ cancer	59 (14.50)	39 (13.73)	20 (16.26)	0.5059
Hematological cancer	216 (53.07)	163 (57.39)	53 (43.09)	0.0079
Transplantation				
No	386 (94.84)	270 (95.07)	116 (94.31)	0.3809
Bone marrow transplantation	8 (1.97)	4 (1.41)	4 (3.25)	
Organ transplantation	13 (3.19)	10 (3.52)	3 (2.44)	
During the hospitalization				
Other infectious diseases	171 (42.01)	104 (36.62)	67 (54.47)	0.0008
Acute renal failure	20 (4.91)	6 (2.11)	14 (11.38)	<0.0001
Use of voriconazole				
No	125 (30.71)	83 (29.23)	42 (34.15)	0.3230
Yes	282 (69.29)	201 (70.77)	81 (65.85)	
Use of steriod				
No	76 (18.67)	69 (24.30)	7 (5.69)	<0.0001
Yes	331 (81.33)	215 (75.70)	116 (94.31)	

SD: standard deviation. IQR: inter-quartile range. MV: mechanical ventilator

Of 407 patients the overall case fatality rate was 30.22% with female slightly higher then male (32.67% compared to 28.79%). Compared to patients who survived, patients who died during the hospitalization were older, more likely to be intubated, admitted to ICU, had a longer ICU stay, and underwent bronchoscope study. They also had a greater prevalence for underlying DM, COPD, and were more likely to have acute renal failure and other infectious diseases during hospitalization. Steroid was used more frequently in the in-hospital death group as compared to patient who survived.

**Fig 1:**
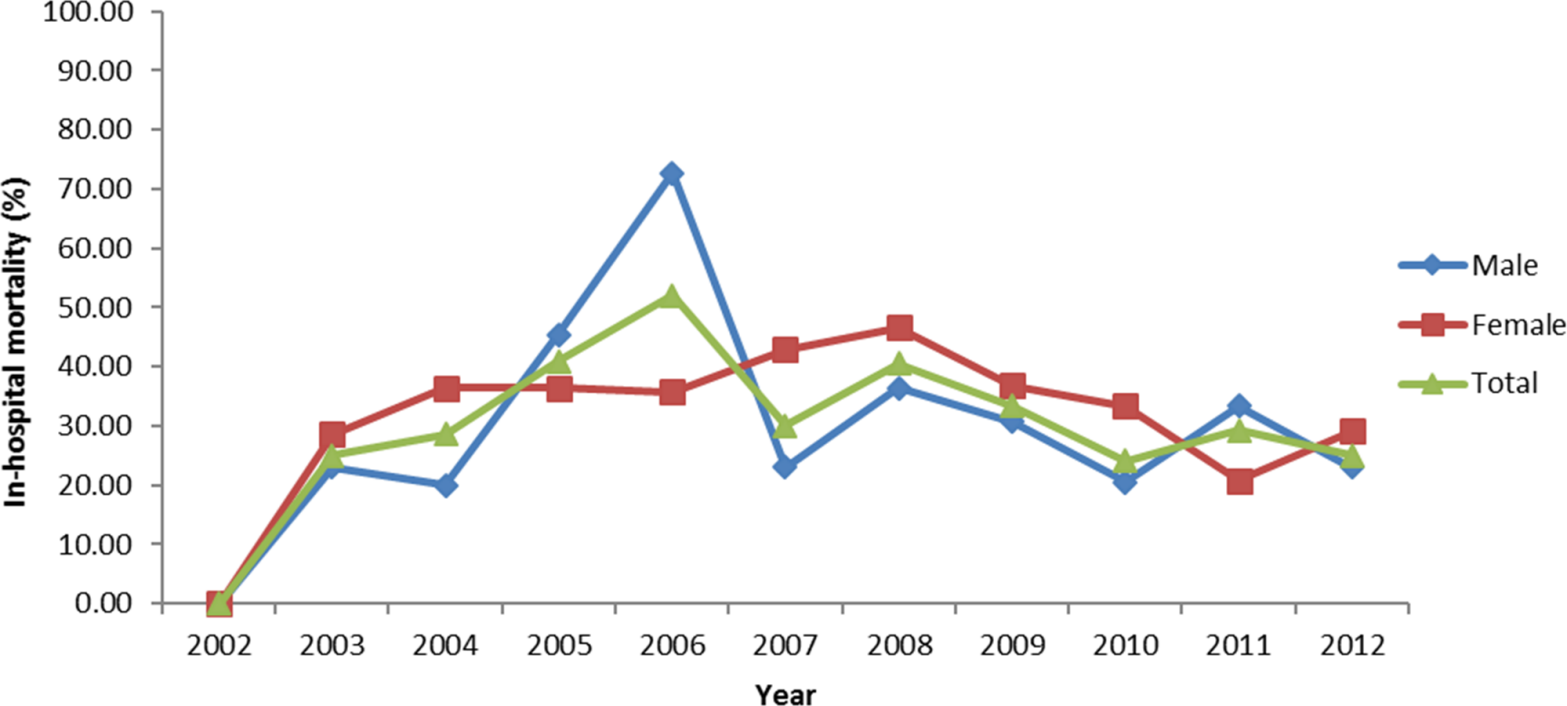
Relationship of mortality in different sex over time.

**Fig 2:**
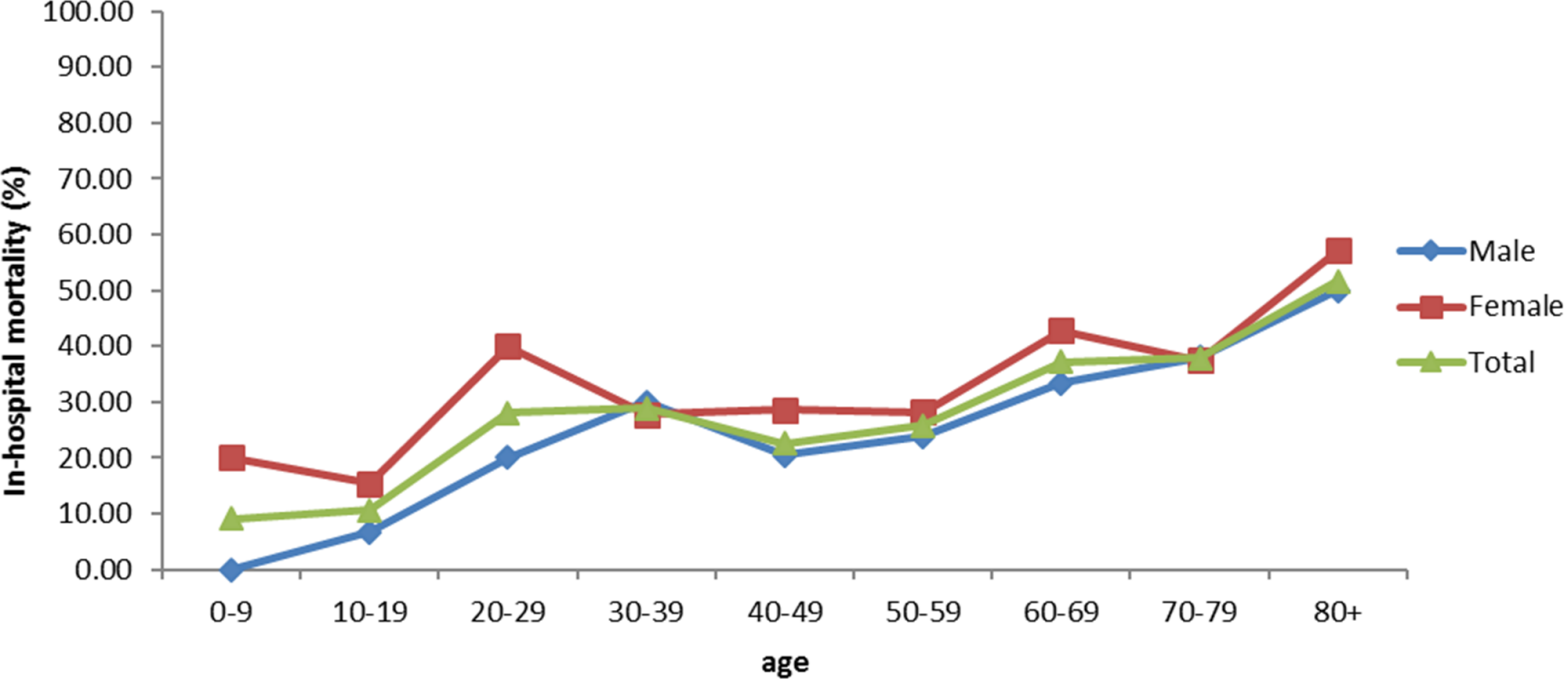
Relationship of mortality with age in different sex.

Fig 1 shows the trend in mortality over time. For both genders, the in-hospital case fatality rate increased since 2002, peaked in 2006 and 2008, then declined over time with an overall in-hospital mortality of 25% in 2012. Of note, there were more cases identified from 2007 to 2012 compared to the period from 2002 to 2006 (307 versus 100). Fig 2 shows the relationship between mortality and age. The proportion of deaths increased with age, regardless of sex. Furthermore, females had a higher mortality compared to males across almost all age groups. Table 2 shows the results of the logistic regression using both univariate and multivariate models. In the multivariate model, female gender, older age (≥ 65 years old), intubation, bone marrow transplantation, acute renal failure, other infectious diseases and steroid use were identified as predictive factors for mortality in patients with IPA.

**Table 2:** Odds ratio and 95%CI for in-hospital mortality in patients with invasive aspergillosis pneumonia.

	Univariate analysis		Multivariate analysis	
Variables -Number (%)	OR	95%CI	OR	95%CI
Gender				
Female	Reference	—	Reference	—
Male	0.87	0.56–1.36	0.49	0.25–0.96
Age, years				
0–18	Reference	—	Reference	—
19–49	3.56	1.02–12.47	1.50	0.33–6.84
50–64	3.48	0.99–12.26	1.74	0.39–7.81
65+	7.89	2.30–27.07	5.55	1.23–24.93
GM test	1.21	0.77–1.91	0.92	0.49–1.74
Use of intubation and MV	15.36	9.15–25.77	25.78	12.59–52.81
Comorbidity				
DM	1.74	1.04–2.94	2.01	0.93–4.37
ESRD	1.31	0.43–4.00	2.93	0.37–23.42
Liver cirrhosis	0.57	0.21–1.55	0.52	0.12–2.33
COPD	2.21	1.15–4.26	2.11	0.77–5.79
Asthma	0.90	0.37–2.22	0.33	0.09–1.17
Bronchiectasis	0.83	0.29–2.35	0.69	0.17–2.83
Tuberculosis	0.88	0.42–1.82	0.82	0.27–2.46
AIDS	2.36	0.33–16.96	4.73	0.24–91.98
Autoimmune disease	1.41	0.63–3.18	0.53	0.16–1.82
Cancer				
Solid organ cancer	1.17	0.64–2.11	1.73	0.73–4.08
Hematological cancer	0.58	0.38–0.89	1.89	0.89–4.00
Transplantation				
No	Reference		Reference	
Bone marrow transplantation	2.36	0.58–9.60	16.09	3.16–82.04
Organ transplantation	0.71	0.19–2.62	0.38	0.04–3.52
During the hospitalization				
Other infectious diseases	2.03	1.32–3.12	2.54	1.38–4.68
Acute renal failure	6.04	2.26–16.13	6.65	1.4–31.55
Use of voriconazole				
No	Reference	—	Reference	—
Yes	0.78	0.50–1.23	0.97	0.50–1.90
Use of steriod				
No	Reference	—	Reference	—
Yes	5.15	2.29–11.58	3.25	1.18–8.97

## Discussion

The present study illustrates the trend in in-hospital mortality among patients with IPA over a period of 11 years. Several risk factors for in-hospital mortality were also identified, including female gender, old age, mechanical ventilation, bone marrow transplantation, acute renal failure, other infectious diseases, and use of steroid.

Population-based epidemiological reports evaluating IA are not common, and risk factors for mortality have been poorly identified. Many of the research have been carried out at single institutions with limited case numbers and in some cases, studies targeted only patients with hematological disorders [17, 18]. Therefore, it is difficult to generalize the results of these studies, and a clear understanding of the epidemiology of aspergillosis is lacking.

This nationwide study in Taiwan identified several factors relevant to patient outcomes, which can be used as a guide for clinical judgment and future studies. Age is a significant factor for predicting mortality. Fig 2 shows that the in-hospital mortality rate increases with age. In the multivariate regression model, the odds ratio for mortality was as high as 5.55 for patients aged 65 years and over compared to patients less than 19 years old. This result is consistent with a previous international multicenter epidemiological study by Taccone et al [1], though other studies did not identify age as a risk factor for mortality. One possible explanation for this discrepancy is that these studies included patients from single healthcare institutions who were less representative than the patients in our nationwide study and multicenter research. In our study, female patients were more likely to die from IPA; this finding has never been observed in previous studies. The reason for this novel finding is unknown, and additional studies are required to further substantiate and explain this observation.

Underlying comorbidities may affect the outcome of patients with IA. Previous studies have demonstrated that bone marrow transplantation, diabetes and neutropenia are associated with an increased risk of mortality [1, 16, 19]. In the present study, bone marrow transplantation was the only comorbidity found to be predictive of mortality. This inconsistency may be a result of different study designs, disease definition, and the quality of patient care. Additional large-scale reports are necessary to better demonstrate this correlation.

Mechanical ventilation, acute renal failure and other infectious diseases were also identified as predictive factors for mortality. In line with previous reports [15, 19, 20], use of a mechanical ventilator was the single most important risk factor for mortality in our study population. Feasible comparisons could not be made with other similar studies that did not include mechanical ventilation or respiratory failure in their analyses. The correlation between mechanical ventilation and mortality is not surprising since the need for a ventilator signifies severe lung involvement, indicating a poor prognosis. Similarly, acute renal failure and other infectious diseases during hospital stay are complications that may occur in patients with severe fungal infection. The associations between these two conditions and increased risk of death have been illustrated in studies investigating invasive aspergillosis [14, 21, 22].

The case fatality rate was 30.22%, which was similar to the results of two large-scale registry studies [23, 24]. Over the years, the trend in in-hospital mortality fluctuated and decreased in the latter period of the study. The introduction of new diagnostic tools and anti-fungal agents may have contributed to this reduction in mortality. However, even though the mortality gradually decreased, it remained high. The results suggest that, in addition to the available diagnostics and medications, further research and better patient care models are needed to improve the outcome of patients with IPA. As a nationwide study that included a large set of patients with IPA over a period of 11 years, the results are likely to be representative of the Taiwanese population.

However, there are some limitations. First, because the data were retrieved from a national health insurance registration system, information on clinical symptoms and culture results are not available. This did not allow us to include and classify patients on the basis of the definition proposed by the European Organization for Research and Treatment of Cancer/Invasive Fungal Infections Cooperative Group and the National Institute of Allergy and Infectious Diseases Mycoses Study Group Consensus Group [25]. We acknowledged this weakness and attempted to overcome it. Following Kim et al., we used strict inclusion criteria, i.e. hospitalized patients receiving at least 3 days of anti-mold medications, which we believe covers the patients most likely to have IPA. Second, the lack of clinical data also made it difficult to account for disease severity by using standardized scales such as SOFA score, which may also influence the results of risk factor analysis for mortality. Third, we did not evaluate causes of death, and we could not exclude the possibility that some mortality may have been due to the concomitant disease, such as co-infections.

In conclusion, we identified several factors that were associated with death among patients with IPA. Risk factors for mortality included old age, female gender, mechanical ventilation, bone marrow transplantation and steroid use. The development of acute renal failure and other infectious diseases during hospitalization were also indicators for in-hospital death. The trend in mortality over the study period was also illustrated. Further research on better patient management is needed to reduce mortality and the clinical burden of IPA.
